# Cost of dengue outbreaks: literature review and country case studies

**DOI:** 10.1186/1471-2458-13-1048

**Published:** 2013-11-06

**Authors:** Hans-Christian Stahl, Vicki Marie Butenschoen, Hien Tinh Tran, Ernesto Gozzer, Ronald Skewes, Yodi Mahendradhata, Silvia Runge-Ranzinger, Axel Kroeger, Andrew Farlow

**Affiliations:** 1 Global Health Task Force, Freiburg University Medical Center, Freiburg im Breisgau, Germany; 2 Medical Faculty Mannheim, Mannheim Institute of Public Health, Mannheim, Germany; 3 Center for Tropical Medicine, Oxford University Clinical Research Unit Vietnam, 764 Vo Van Kiet P1Q5, Ho Chi Minh City, Vietnam; 4 Facultad de Salud Pública y Administración, Universidad Peruana Cayetano Heredia, San Martín de Porres, Lima, Perú; 5 Ministerio de Salud, Dirección General de Epidemiología, Santo Domingo de Guzmán, República Dominicana; 6 Center for Tropical Medicine, Faculty of Medicine, Gadjah Mada University, Yogyakarta, Indonesia; 7 Consultant to the Special Programme for Research and Training of Tropical Diseases (TDR) at the World Health Organization (WHO), Geneva, Switzerland; 8 Special Programme for Research and Training of Tropical Diseases (TDR) at the World Health Organization (WHO), Geneva, Switzerland; 9 School of Tropical Medicine, Liverpool, United Kingdom; 10 Department of Zoology, Spatial Ecology & Epidemiology Group, University of Oxford, Oxford, United Kingdom

**Keywords:** Dengue outbreaks, Dengue surveillance, Cost of dengue outbreaks

## Abstract

**Background:**

Dengue disease surveillance and vector surveillance are presumed to detect dengue outbreaks at an early stage and to save – through early response activities – resources, and reduce the social and economic impact of outbreaks on individuals, health systems and economies. The aim of this study is to unveil evidence on the cost of dengue outbreaks.

**Methods:**

Economic evidence on dengue outbreaks was gathered by conducting a literature review and collecting information on the costs of recent dengue outbreaks in 4 countries: Peru, Dominican Republic, Vietnam, and Indonesia. The literature review distinguished between costs of dengue illness including cost of dengue outbreaks, cost of interventions and cost-effectiveness of interventions.

**Results:**

Seventeen publications on cost of dengue showed a large range of costs from 0.2 Million US$ in Venezuela to 135.2 Million US$ in Brazil. However, these figures were not standardized to make them comparable. Furthermore, dengue outbreak costs are calculated differently across the publications, and cost of dengue illness is used interchangeably with cost of dengue outbreaks. Only one paper from Australia analysed the resources saved through active dengue surveillance. Costs of vector control interventions have been reported in 4 studies, indicating that the costs of such interventions are lower than those of actual outbreaks. Nine papers focussed on the cost-effectiveness of dengue vaccines or dengue vector control; they do not provide any direct information on cost of dengue outbreaks, but their modelling methodologies could guide future research on cost-effectiveness of national surveillance systems.

The country case studies – conducted in very different geographic and health system settings - unveiled rough estimates for 2011 outbreak costs of: 12 million US$ in Vietnam, 6.75 million US$ in Indonesia, 4.5 million US$ in Peru and 2.8 million US$ in Dominican Republic (all in 2012 US$). The proportions of the different cost components (vector control; surveillance; information, education and communication; direct medical and indirect costs), as percentage of total costs, differed across the respective countries. Resources used for dengue disease control and treatment were country specific.

**Conclusions:**

The evidence so far collected further confirms the methodological challenges in this field: 1) to define technically dengue outbreaks (what do we measure?) and 2) to measure accurately the costs in prospective field studies (how do we measure?). Currently, consensus on the technical definition of an outbreak is sought through the International Research Consortium on Dengue Risk Assessment, Management and Surveillance (IDAMS). Best practice guidelines should be further developed, also to improve the quality and comparability of cost study findings. Modelling the costs of dengue outbreaks and validating these models through field studies should guide further research.

## Background

Dengue is geographically a fast-spreading disease (WHO 2012) [1]. Outbreaks hit the economy and health systems of endemic countries with variable epidemiologic magnitude. Resources used for the prevention and treatment of dengue are not available for alternative uses in health systems or in the economy as a whole. Dengue is in competition with other diseases for the limited resources available, especially in endemic countries with dengue-outbreaks, where too there are efforts to bring the costs of dengue interventions within the reach of less wealthy populations. Health technologies available to reduce the consumption of resources used to prevent and treat dengue are therefore of high interest, since resources saved can be allocated to other diseases and sectors of the economy. Investigations on the efficacy of health technologies to combat dengue are nowadays always supplemented by health economic analyses.

As a first step, this paper reviews available literature on cost of dengue outbreaks worldwide. As a second step, country case studies are used to gather first-hand evidence on the direct costs of dengue outbreaks in 2011 in four selected countries: two countries in the Americas (Peru and Dominican Republic) and two countries in South East Asia (Vietnam and Indonesia). The ultimate aim, which is not part of this technical report, is to appraise the efficiency of improved surveillance.

Beatty et al. (2011) [2] reviewed the methodology used in the health economic literature on dengue from 1966 to 2009. Although the publication did not focus on reviewing the actual costs of dengue, it classified systematically the available health economic dengue literature. Shepard et al. (2011) [3] estimated the cost of the dengue illness in all the countries in The Americas combined to be about 2.1 billion US$ on average per year. Comparable figures for the Asian region were missing at the time of literature review, but have recently been calculated by Shepard et al. (2013) [4]. The present paper aims at reviewing all available published evidence on cost of dengue outbreaks independently of the methodology employed to calculate these costs. Despite the absence of a unanimous technical definition of a dengue outbreak, the paper aims to introduce a distinction between cost of dengue outbreaks and cost of dengue illness studies. Cost-of-illness implicitly calculates the opportunity cost of an illness versus a theoretical situation where the disease has been eliminated.

The rationale of this research is to provide ministries or official institutions in dengue-endemic countries with evidence on the costs of dengue outbreaks, so as to guide them on the expected returns on their investments in surveillance systems tailored to identify, respond to and stop potential dengue outbreaks at an early stage. The underlying assumption was that by showing the costs of a dengue outbreak, governments would be more inclined to put resources into outbreak prevention, early outbreak detection and response.

Besides the practical difficulties in ascertaining costs of dengue outbreaks in the field, the costing of dengue outbreaks is problematic in several other respects: i) the health systems and the economy of countries are very different in terms of their health systems’ structures and the resources deployed to combat dengue. ii) there is no unique worldwide dengue outbreak definition [5]; iii) the measurement of how much of an outbreak could be avoided by certain interventions might be ethically and practically impossible to conduct in a prospective field study.

However, the aim of the literature review is to study the publications on costs of dengue, with respect to: A) how these dengue costs have been measured and B) what were the findings in terms of cost of dengue.

## Methods

### Literature review

#### Search strategy

The literature review was conducted in the following databases: the United States National Library of Medicine and the National Institutes of Health Medical Database (PubMed) (1966–2012); the Cochrane Database of Systematic Reviews (CDSR); the World Health Organisation (WHO) library database (WHOLIS) and the Latin American and Caribbean Health Sciences Database (Lilacs) (1967–2012); Econlit; and the library of the Pan American Health Organization (PAHO). The free text search terms “cost” and “dengue” were used. All citations up to April 1st 2012 were included, irrespective of language or publication year.

We identified 220 citations out of which we selected 56 eligible abstracts according to the following criteria: A) analysed cost; B) provided detailed methods; C) included data analysis. Out of these selected abstracts we identified 12 duplicates and 3 papers that were not accessible to us. Finally, 41 citations were selected for full text review. Dengue cost studies were defined as analyses identifying, quantifying and valuing in monetary terms those resources used (direct medical, direct non-medical, and indirect) at any stage of dengue disease prevention or control, on any geographical level (region, country, continent, world) from any perspective (patient, relatives, government, provider or societal) at any level of intervention (vector, cases) and disease severity, including or excluding assessments of effectiveness of health technologies. The rationale was to collect as much information as possible from different publications, wherever costs were analysed in the context of dengue, with the aim of compiling dengue outbreak costs.

#### Evidence level

The reliability of evidence of the papers was not assessed in a thorough manner within the present review. Based on the Beatty et al. [2] classification scheme we identified the level of evidence of those studies prior to 2009 (Table 1).

**Table 1:** **Evidence score of publications reviewed (see Beatty et al. (2011) **[2]**)**

**Quality score**	**Description**
I	Evaluation of important alternative interventions comparing all clinically-relevant outcomes against appropriate cost measures, and including a clinically-relevant sensitivity analysis
II	Evaluation of important alternative interventions comparing a limited number of outcomes against appropriate cost measurement, but including a clinically-sensible sensitivity analysis
III	Evaluation of important alternative interventions comparing all clinically-relevant outcomes against inappropriate cost measurement, but including a clinically-sensible sensitivity analysis
IV	Evaluation without a clinically-sensible sensitivity analysis
V	Expert opinion with no explicit critical appraisal, based on economic theory
UE	Ungraded evidence

#### Data extraction and synthesis

Two independent authors (VMB and HCS) assessed the full texts. Consensus was achieved about inclusion and exclusion criteria. Of the 41 studies, general information was retrieved on the following categories: First Author, Publication, Publication Year, Countries, Cities, Study Period, Intervention(s), Control, Randomized, Perspective, Outcome, Subjects, Inclusion Criteria, Exclusion Criteria, Total Cases, Cost Type, Cost Year, Currency, Cost Horizon, Costs, Effect Type, Effect Year, Effect Horizon, Effects, Remarks. In a second-round review, studies were excluded that met the following criteria:

1. reviews of already published original publications

2. analyses of the burden of disease in non-monetary terms.

Publications were then classified in different groups depending on the aim of cost collection:

A. cost of dengue illness or cost of dengue outbreaks

B. cost of intervention

C. cost-effectiveness of intervention

We analysed also studies that included cost of interventions, since our final interest is to assess the cost-effectiveness of surveillance systems. The flow chart of the literature review process is represented in Additional file 1: Figure S1.

The literature review was protocoled according to the PRISMA checklist given in Additional file 2: Table S1.

### Country case studies on costs of dengue outbreaks

#### Collection of costs

The perspective taken in this field-study cost collection was societal, based on a mixture of micro- and macro-costing data collection. The aim was to calculate the average treatment costs of a dengue patient based on a micro-costing approach, and multiply the costs by the number of cases during the outbreak. Indirect costs were not collected because, in the context of this study, the interest was focussed on the costs for health services and their administration. Expansion factors were not used. Surveillance systems might under- or over-report the total number of cases. These numbers may be corrected by multiplying reported cases by an expansion factor (EF).

Resource consumption in medical care was collected in hospital settings by contacting interviewers in four dengue-endemic countries, which had experienced recent dengue outbreaks: Dominican Republic, Peru, Indonesia, and Vietnam. The countries were selected by the Special Programme for Research and Training in Tropical Diseases at the World Health Organization in Geneva (TDR-WHO) and by the WHO Regional offices PAHO and SEARO. In each country a data collector was hired with background in public health and some training in economics. The country interviewers received the research protocol, detailed instructions about how to proceed, and a data collection form for costing individual patients. In each country, the interviewers collected data from 5 to 10 dengue patients with the pre-defined form in different health service settings and categories: outpatients, children in-patients, adult in-patients, children in intensive care units, adults in intensive care units. The interviewers collected data through document analysis (clinical histories of patients in the different treatment categories) and interviews with key informants. The latter included: dengue clinicians, to establish the typical treatment and required resources for a dengue patient; laboratory staff, to complement the information of the clinician; pharmacy staff, to establish the cost of each item; chief entomologist or vector control manager and staff in the communications department.

#### Data analysis

Through the above step-wise approach, the typical treatment and average treatment costs per patient in the different patient categories were established. On the basis of the patient samples, averages were calculated for the following categories: laboratory; technical services; drugs; consumables; fees; and other resources. The sum of these categories was the total treatment cost per patient derived from our sample for the respective treatment setting. The average direct medical cost per patient without personal costs was derived by multiplying the total costs per patient in each treatment setting by the number of patients during the outbreak in each treatment setting, summed up over the different settings, and dividing by the total number of reported patients during the outbreak. Additionally, the interviewers collected information from national sources on expenditures for vector control, surveillance, information, education and communication during the most recent outbreak.

#### Ethical aspects

Exemption from ethical approval was granted by WHO Regional Offices based on the fact that no direct individual information was collected. However, each respondent signed an informed consent form.

## Results

### Systematic literature review

#### Publications on cost of dengue illness and cost of dengue outbreaks (group A)

In total 17 publications met the inclusion criteria. All but 2 publications collected primary data, and most studies took a societal perspective without indicating it explicitly. Of the 17 publications, 5 [6-10] intentionally narrowed the perspective to the impact of dengue illness at the household level. Of these household studies, two were conducted in Cambodia, two in Thailand and one in Vietnam. The average cost of dengue illness per patient ranged from 16.6 US$ [6] for children at primary school to 24 US$ per family in Thailand [7], from 31.5 US$ for households with children aged <15 years [10] to 32 US$ for households treated in public and private hospitals in Cambodia [8] and 61.3 US$ for children with dengue haemorrhagic fever (DHF) in Vietnam [9].

Out of the 17 publications, 2 used modelling, with a mixture of primary and secondary data, to assess the cost of illness in India [11], and for all the countries in the Americas [3]. Both of these included costs that were direct, indirect, and due to fatal cases, but not costs related to vector control, surveillance, and information, education and communication. The total economic dengue burden in India was estimated to be 27.4 million US$ compared to 2.1 billion US$ in the Americas.

Only one study [12], conducted in Cuba, was designed as a prospective study comparing, in a non-endemic dengue region of Cuba, a period of no transmission with a period of transmission, so as to calculate the incremental cost of a dengue outbreak. The total economic cost per inhabitant per month was around 2.7 US$ in months without transmission versus 6 US$ in outbreak periods. The population and the health care system largely incurred the cost increments, but hardly any of the costs of the vector control programme, which are usually fixed costs. The average cost per hospitalised dengue case was 296.60 US$ in Cuba.

With the exception of the incremental approach in the Baly et al. 2012 study [13], the method chosen to calculate the total cost of dengue outbreaks is to multiply the number of reported cases by an expansion factor (ratio of observed to reported cases) and by the average cost per case (direct and indirect costs) and to add the surveillance, vector control, information, education and communication budgets of the communities and the central governments (where applicable). The terminology ‘cost of dengue outbreak’ and ‘cost of dengue illness’ are often used as synonyms. An unambiguous definition of ‘cost of dengue outbreak’ is currently missing, although Baly et al. 2012 [13] suggested an alternative way to calculate incremental outbreak costs.

Out of the 17 publications, 9 covered the cost of dengue illness with differing degrees of evidence regarding their primary data collection: Cambodia [14], Cuba [15,16], Venezuela [17], Panama [18], Australia [19], Thailand [20], Americas & Asia [21] and Puerto Rico [22]. Total cost of dengue illness for Cambodia [14] ranged from 3.3 million US$ to 14.4 million US$ depending on the year considered (2007–2008) and the cost of dengue cases ranged from 27 US$ to 75 US$, including direct and indirect costs. In Cuba, total cost of dengue illness ranged from 10.3 million US$ in 1997 [16] to 103.2 million US$ in 1981 [15]. In Thailand, the 1994 total dengue cost was estimated to be 12.6 million US$. The indirect and direct cost per dengue case in Bangkok amounted to 161.5 US$ for an adult and 118.3 US$ for a child. In Panama the 2005 outbreak induced total costs of 16.9 million US$ (5.2 US$ per capita) including direct and indirect costs and government spending on dengue control efforts [18]. A publication on the cost of dengue illness between 1997–2003 in Venezuela reported total costs of 1.4 million US$ [17] for that period. The first study form 1977, of cost of dengue illness in Puerto Rico estimated total costs to vary between 6 to 15.6 million US$ [22] depending on the attack rate of mosquitoes. Suaya et al. [21] estimated the aggregate annual cost of dengue illness in the Americas, covering Brazil (135.2 million US$), El Salvador (1.7 million US$), Guatemala (1.2 million US$), Panama (0.9 million US$) and Venezuela (10.2 million US$) to be about 149.3 million US$ based on incidence for the years 2001 to 2005 and primary data collected from 1,160 patients. Suaya [21] estimated the aggregate annual cost of dengue illness for Asia, covering Cambodia (2.8 million US$), Malaysia (38.2 million US$) and Thailand (47.8 million US$), to be about 88.8 million US$ based on the 2001–2005 reports and on primary data collected for 535 patients in total. For Australia, Canyon [19] estimated total annual dengue illness costs to be about 2.7 million US$ per year since 1990.

#### Publications on cost of interventions (group B)

We identified 4 studies calculating the cost of interventions: one in Vietnam on community-based strategies against the vector Aedes aegypti [23], one in Venezuela and Thailand on the cost of routine aedes aegypti control and insecticide-treated curtain implementation [24], one in Australia on costs of cutting mosquito surveillance budgets [25] and one in Brazil on the direct costs of dengue prevention and control programs [26].

For Australia, Vazquez-Prokopec et al. [25] showed the effect of delayed response to dengue outbreaks of 4 to 6 weeks, which would result in 86 times (or 13 million US$ in 2003) and 346 times (or 382 million US$ in 2009) higher dengue illness costs versus a scenario with active surveillance and response within 2 weeks. Their calculations suggest the value of an active and fast dengue surveillance and response system. The calculations of Baly et al. [12] of the costs of control programs in one state in Venezuela (0.38 million US$) and in one province in Thailand (0.08 million US$) and of Kay et al. [23] for Vietnam also suggest the low cost of dengue control measures compared to the cost of uncontrolled dengue outbreaks. Taliberti et al. [26] estimate the cost of dengue prevention and control in Sao Paolo in 2005 to be 12.5 million US$ (or 1.14 US$ per capita).

#### Publication on cost-effectiveness of interventions (group C)

Three publications out of the 9 in group C investigated the cost-effectiveness of a dengue vaccine. Carrasco et al. [27] investigated the cost-effectiveness of future vaccination programs in Singapore, whereas Lee et al. [28] assessed the economic value of a dengue vaccine in Thailand, and finally Shepard et al. [29] evaluated the cost-effectiveness of a paediatric dengue vaccine.

Six publications assessed the cost-effectiveness of interventions aimed at improving dengue vector control. Alphey et al. [30] assessed the cost-effectiveness of genetics-based sterile insect methods for vector control. Suaya et al. [31] investigated the cost-effectiveness of larviciding campaigns in Cambodia. Baly et al. [24] compared vertical versus community-based approaches in Cuba. McConnell et al. [32] focused on control programs in Puerto Rico, whereas Orellano et al. [33] analysed the cost-benefit of control programs versus no control in Argentina. Luz et al. [34] developed an economic model to assess dengue vector control strategies. The modelling methods used for assessing the economic value of vaccine intervention and the cost-effectiveness of vector control programs might lead to future research on the cost-effectiveness of national surveillance systems.

The complete extraction matrix of the selected and reviewed literature is given in the Additional file 3: Table S2.

### Country case studies

#### Vietnam

**Figure 1:**
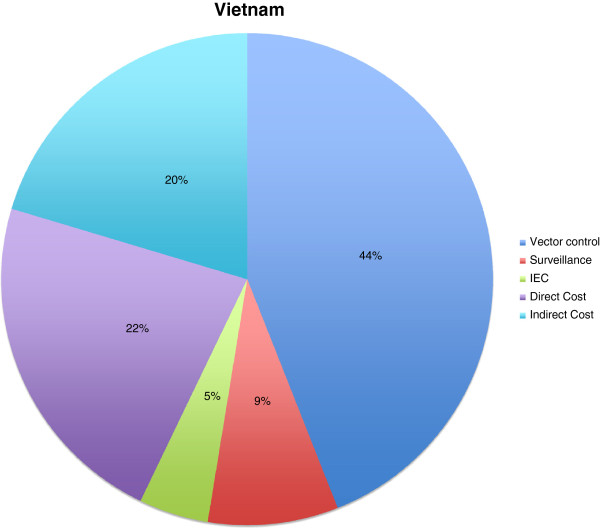
**Cost components during dengue outbreaks in Vietnam.** All in US$ of the year 2011; Vector control: 5,285,280 US$ (44%); Surveillance: 1,029,600 US$ (9%); IEC: 549,120 US$ (5%); Direct cost: 2,700,138 US$ (22%); Indirect cost: 2,447,000 US$ (20%).

For Vietnam, a sample of patient records was collected in one hospital, differentiating four groups of patients comprising outpatients, those treated in the general ward subdivided into adults and children (<15 years), and patients in intensive care units (in this case, no distinction between adult and children was made as there was no major cost difference). The analysis was conducted on the basis of 2012 Vietnam Dong (VND) and an exchange rate of 20,833 (VND/US$) on June 13th, 2012. The analysis for Vietnam showed that direct medical costs were approximately: per case without staff costs 43 US$; per outpatient 27 US$; per adult general ward inpatient 53 US$; per child general-ward inpatient 46 US$; per ICU patient 108 US$. Harving et al. (2007) [9] found similar direct costs (32 US$, that would roughly match our findings if accounting for inflation between 2007 to 2012) and established a ratio of direct versus indirect costs (US$ 32 direct cost to US$ 29 indirect costs). We applied the same ratio to our findings. Other cost components in Vietnam were: government budgets for vector control 5,285,280 US$, or 44%; surveillance 1,029,600 US$, or 9%; and IEC (information, education and communication, 549,120 US$, or 5%. When adding these to the direct (2,700,138 US$ or 22%) and indirect cost (2,447,000 US$ or 20%), we estimated that the outbreak costs for Vietnam in 2011 were around 13 million US$ with 69,680 dengue patients reported (Figure 1).

#### Indonesia

Indonesia collected costs for six patient groups: a typical dengue outpatient (separately for adult and child); a typical general-ward patient (separately for adult and child), and a typical intensive-care-unit patient (separately for adult and child). The exchange rate used was 9,523 Rupiah/US$ on August 15th, 2012 (Table 2).

**Table 2:** Typical direct medical cost of a dengue patient, by treatment setting without personal cost, in Indonesia

**Treatment setting**	**Typical cost (US$)**	**Typical cost (Rupiah)**
Outpatient (<15 years)	50,080	5
Outpatient (>15 years)	52,870	6
General ward (<15 years)	958,715	101
General ward (>15 years)	2,353,683	247
ICU (<15 years)	17,637,762	1,852
ICU (>15 years)	5,618,929	590

**Figure 2:**
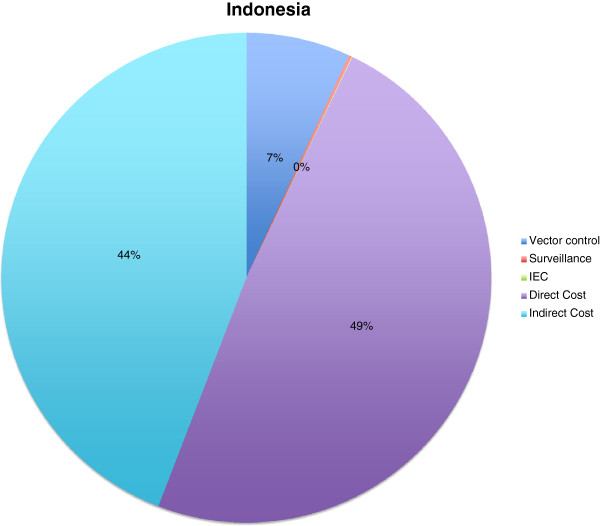
**Cost components during dengue outbreaks in Indonesia.** All in US$ of the year 2011; Vector control: 465,676 US$ (7%); Surveillance: 13,722 US$ (0.2%); IEC: 2,927 US$ (0.04%); Direct cost: 3,288,168 US$ (48.7%); Indirect cost: 2,979,902 US$ (44%).

The number of dengue patients during the last outbreak in Indonesia in 2011 could not be estimated because there was no official definition of an outbreak with a defined start and end-point, and only dengue haemorrhagic fever patients were reported. However, in the recent Jakarta outbreak 17,776 patients were reported. Although no reliable estimate of the distribution of dengue patients in the different treatment settings listed in the table above could be derived, we used the proportions of outpatients versus general-ward inpatients versus intensive care patients established in Vietnam, that was 55% versus 35.1% versus 9.9% respectively. We obtained an estimate of total treatment costs (3,288,200 US$ or 49%) in the Jakarta outbreak, which was the sum of 53,800 US$ for outpatients, 1,085,700 US$ for general ward patients and 2,148,700 US$ for intensive care patients. However, an estimate of public sector cost in the outbreak area was available: Surveillance was estimated to amount to 13,722 US$ (or 0.2%), vector control to 465,676 US$ (or 6.8%) and information, education and communication to 2,927 US$ (or 0.04%). When applying the findings in Vietnam (where indirect costs were around 90% of the direct cost) to Indonesia we obtain indirect costs of 2,979,902 US$ or 44% (Figure 2).

#### Peru

**Figure 3:**
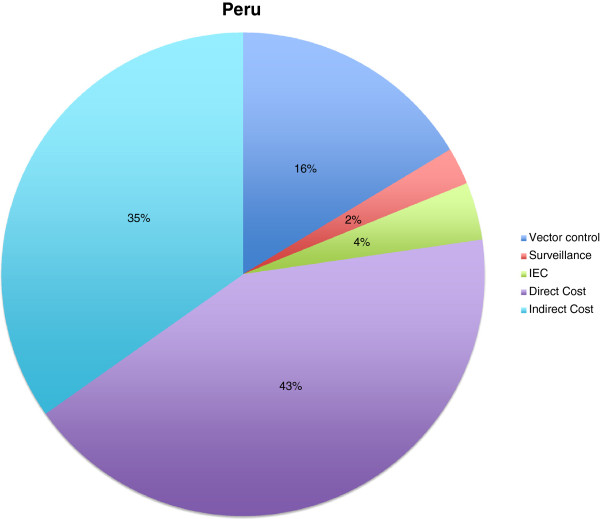
**Cost components during dengue outbreaks in Peru.** All in US$ of the year 2011; Vector control: 738,701 US$ (16%); Surveillance: 112,024 US$ (2%); IEC: 173,842 US$ (4%); Direct cost: 1,917,791 US$ (43%); Indirect cost: 1,569,102 US$ (35%).

Resource utilization for the “typical” dengue patient, with indication of the probability of item usage, was collected in four different hospitals in Peru: Hospital de Apoyo Iquitos, Hospital Regional Iquitos, Hospital Tarapoto and Hospital Solidaridad. We calculated cost in Nuevo Sol (PEN) 2012 and used an exchange rate (PEN/US$) of 2.678 on June 13th, 2012. The number of total cases in Iquitos came to 24,506, of which 21,740 were outpatients, 2,635 were inpatients in general wards and 131 were intensive-care-unit patients. The number of cases in each category was based on dengue without or with warning signs, and severe dengue. The proportion of adults (>15 years) was estimated to be 40% in outpatients, 50% in general wards and 30% in the ICU setting. We found that direct treatment costs per patient without staff cost amounted to 78 US$ (in 2012 US$) in Peru. Based on Shepard et al. (2011) [3], we assumed a proportion of 55 to 45 per cent direct-to-indirect costs in Peru and calculated total direct and indirect cost of 142 US$ per patient. This corresponds to 37% of the amount of 384 US$ calculated by Shepard et al. (2011) [3], which might include other cost items, such as staff costs and direct non-medical costs. Surveillance, vector control and IEC budgets were collected for the study area in Peru (region San Martin and Loreto). The government expenditures were: for vector control 738,701 US$, or 16%; for surveillance 112,024 US$, or 2%; and for IEC 173,842 US$, or 4%. Adding this to the direct (1,917,791 US$ or 43%) and indirect cost (1,569,102 US$ or 35%), we estimated that the total cost of the Iquitos outbreak in 2011 came to around 4.5 million US$ (Figure 3).

#### Dominican Republic

**Figure 4:**
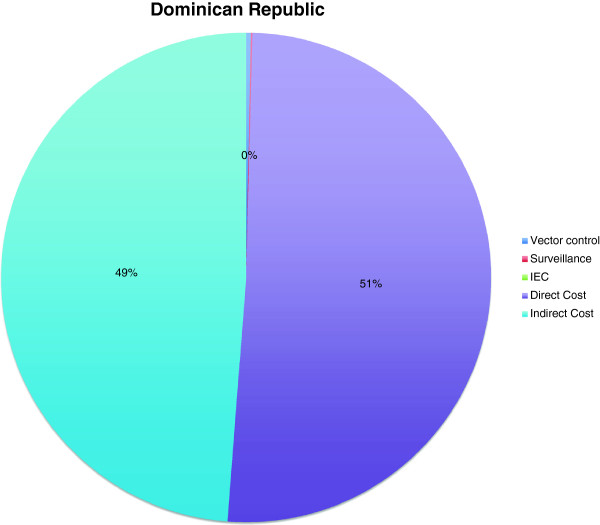
**Cost components during dengue outbreaks in Dominican Republic.** All in US$ of the year 2011; Vector control: 9,165 US$ (0%); Surveillance: 2,550 US$ (0%); IEC: NA; Direct cost: 1,424,007 US$ (51%); Indirect cost: 1,367,047 US$ (49%).

In the Dominican Republic 12,171 dengue patients were reported during the outbreak in 2011, of which 292 were outpatients, 11,864 were general-ward patients and 15 were intensive-care patients. In the general ward 68% of patients were adults, and 32% were children (<15 years). The exchange rate used for the analysis was 39.03 DOP/US$ on June 17th, 2012. For the Dominican Republic, hospital records of 5 outpatients, 9 adult general-ward patients, 28 child general-ward patients, and 15 ICU patients were analysed in terms of resource identification, quantification and valuation. The average direct cost per case without personal costs amounted to 117 US$ in 2012. Shepard et al. (2011) [3] obtain a ratio of indirect to direct cost of 96% for the Dominican Republic. The corresponding indirect cost estimation based on our direct cost estimate is 112 US$. Shepard et al. (2011) [3] obtained a weighted average estimation of total costs of 430 US$ per dengue case based on their economic model. The cost we obtained for government spending on vector control was a total of 9,165 US$ per year on average over the years 2009 to 2012. Cost for collecting, storing, analysing and communicating surveillance data amounted to 2,550 US$. Our total cost estimate was around 2.8 million US$ (Figure 4).

## Discussion and conclusions

### Evidence from the systematic literature review

#### Limitations and comparability of publications

This review included only publications in the given databases within the given time period: 1966 to April 1st 2012. Unpublished data, evaluations, dissertations and books were not included. Publications in Spanish, Portuguese and English were included.

The comparability of the reviewed studies was restricted due to variations in the perspective taken to collect costs, the definition and reporting of costs, and the inclusion or exclusion of certain cost items within a micro-costing approach. Finally, precise details on the year of cost accounting (year of prices), the exchange rate used and the transformations conducted to calculate costs (discounting, inflation adjustment) were missing. The Asian countries had been neglected in terms of methodologically comparable cost of illness studies as produced by Shepard et al. [3] for the Americas. Sensitivity analyses would increase the validity of produced cost figures.

Moreover, the calculation of dengue costs posed technical problems. Under- and over-reporting of dengue cases made the exact number of dengue cases uncertain, even after correcting with an expansion factor. For example the average expansion factor of 2.3 for the Americas had a range of 1.4 to 3.3 in a hospitalized setting and an average of 15 and a range of 9 to 28 in an ambulatory setting. Moreover the average cost per dengue case varied according to country, treatment (ambulatory, hospitalized) and geographical (urban, rural) setting, patient age and hospital type (private, public) and therefore should be calculated as a weighted average, in those situations when the data for each setting is available.

Average total costs per case for all the countries in the Americas based on the costs collected and modelled by Shepard et al. 2011 [3] showed the important variations in costs between countries studied. Average costs per case in the Americas was given as 571 US$, with a 95% confidence interval of 362 to 752 US$, illustrating the important variation between countries. On average, the share of indirect costs to total costs amounts to 60% (40% direct costs), but there is also large variation between countries in the Americas. Moreover, depending on the year considered, the cost of dengue could vary, which was very well illustrated by Shepard et al. 2011 [3], but had been neglected by prior cost of illness studies in dengue, which did not average costs over several successive years. Finally, variations in total costs were substantial depending on the assumed expansion factor, and the share of hospitalized cases or average ambulatory cost per case - varied from 1 to 4 billion US$ in the Americas according to Shepard et al. 2011 [3].

#### Cost and cost-effectiveness of Interventions

Only one paper from Baly et al. [12] addressed the costs of outbreaks explicitly and, to our knowledge, methodologically accurately in a prospective study. All other papers in group A investigated cost of illness with very different methodological approaches, and so faced various technical challenges which need to be addressed in best practice research guidelines. Comparability of the reviewed papers was very limited and, accordingly, a direct comparison between countries was not very meaningful, except in papers with a consistent methodology applied to different countries. Only one paper dealt with modelling the unforeseen cost of cutting the budget for dengue disease surveillance and response, which would be interesting for future modelling research on the cost-effectiveness of improved surveillance systems. Moreover, methods were available and had been applied to interventions in the field of dengue prevention and control to calculate cost-effectiveness ratios.

### Evidence from the country case studies

**Figure 5:**
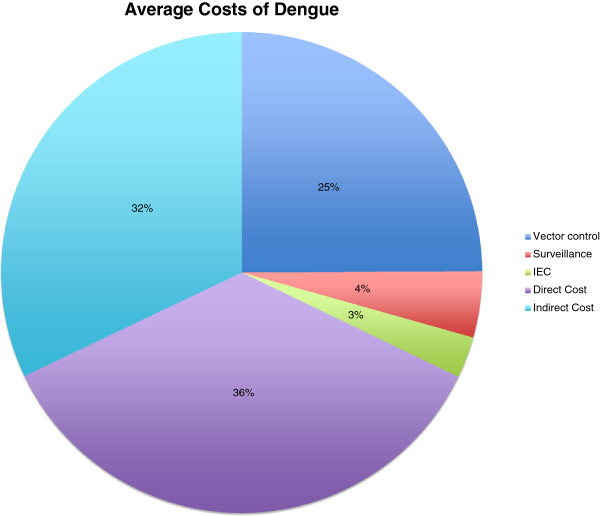
**Total cost components in percent estimated for a recent dengue outbreak in four countries.** Direct costs include laboratory, technical services, drugs, consumables, fees and other resources. Indirect costs include loss of working hours. All in US$ of the year 2011; Vector control: 6,498 US$ (25%); Surveillance: 1,157 896 US$ (4%); IEC: 725, 889 (3%); Direct cost: 9,330,104 US$ (36%); Indirect cost: 8,363,051 US$ (32%).

No data on cost of dengue outbreaks had been published for Vietnam, Indonesia, Dominican Republic and Peru (Figures 1, 2, 3, 4 and 5). Our data provided the first estimates of the potential economic impact of dengue outbreaks in these countries. Further research, in particularly prospective studies, is needed to make these estimates more robust.

## Competing interests

The authors declare that they have no competing interests.

## Authors’ contributions

Conceived and designed the study: HCS, SRR, AK, AF. Performed the data collection: HCS, VMB, HTT, EG, RS, YM. Analysed the data: HCS, VMB, AK, SRR. Wrote the paper: HCS, VMB, AK, SRR, AF. All authors read and approved final version of the manuscript.

## Pre-publication history

The pre-publication history for this paper can be accessed here:

http://www.biomedcentral.com/1471-2458/13/1048/prepub

## Supplementary Material

Additional file 1: Figure S1 PRISMA Flow-chart of review process. Click here for file

Additional file 2: Table S1 PRISMA checklist for literature review. Note: As this manuscript is not a systematic review nor meta-analysis, the entries in the checklist are limited to those items applicable to this manuscript. Click here for file

Additional file 3: Table S2 Extraction matrix of selected and reviewed literature. Click here for file

## References

[B1] WHO | global strategy for dengue prevention and control, 2012–2020 Available from: http://www.who.int/denguecontrol/9789241504034/en/. Accessed 5 May 2013

[B2] Beatty ME Beutels P Meltzer MI Shepard DS Hombach J Hutubessy R Health economics of dengue: a systematic literature review and expert panel’s assessment Am J Trop Med Hyg 2011 84 3 473 488 10.4269/ajtmh.2011.10-0521 21363989 PMC3042827

[B3] Shepard DS Coudeville L Halasa YA Zambrano B Dayan GH Economic impact of dengue illness in the Americas Am J Trop Med Hyg 2011 84 2 200 207 10.4269/ajtmh.2011.10-0503 21292885 PMC3029168

[B4] Shepard DS Undurraga EA Halasa YA Economic and disease burden of dengue in southeast Asia PLoS Negl Trop Dis 2013 7 2 e2055 10.1371/journal.pntd.0002055 23437406 PMC3578748

[B5] Runge-Ranzinger S Horstick O Marx M Kroeger A What does dengue disease surveillance contribute to predicting and detecting outbreaks and describing trends? Trop Med Int Health 2008 13 8 1022 1041 10.1111/j.1365-3156.2008.02112.x 18768080

[B6] Anderson KB Chunsuttiwat S Nisalak A Mammen MP Libraty DH Rothman AL Burden of symptomatic dengue infection in children at primary school in Thailand: a prospective study Lancet 2007 369 9571 1452 1459 10.1016/S0140-6736(07)60671-0 17467515

[B7] Clark DV Mammen MP Nisalak A Puthimethee V Endy TP Economic impact of dengue fever/dengue hemorrhagic fever in Thailand at the family and population levels Am J Trop Med Hyg 2005 72 6 786 791 15964964

[B8] Van Damme W Van Leemput L Por I Hardeman W Meessen B Out-of-pocket health expenditure and debt in poor households: evidence from Cambodia Trop Med Int Health 2004 9 2 273 280 10.1046/j.1365-3156.2003.01194.x 15040566

[B9] Harving ML Rönsholt FF The economic impact of dengue hemorrhagic fever on family level in southern Vietnam Dan Med Bull 2007 54 2 170 172 17521539

[B10] Huy R Wichmann O Beatty M Ngan C Duong S Margolis HS Vong S Cost of dengue and other febrile illnesses to households in rural Cambodia: a prospective community-based case–control study BMC Public Health 2009 9 155 10.1186/1471-2458-9-155 19473500 PMC2696434

[B11] Garg P Nagpal J Khairnar P Seneviratne SL Economic burden of dengue infections in India Trans R Soc Trop Med Hyg 2008 102 6 570 577 10.1016/j.trstmh.2008.02.015 18402995

[B12] Baly A Flessa S Cote M Thiramanus T Vanlerberghe V Villegas E The cost of routine aedes aegypti control and of insecticide-treated curtain implementation Am J Trop Med Hyg 2011 84 5 747 752 10.4269/ajtmh.2011.10-0532 21540384 PMC3083742

[B13] Baly A Toledo ME Rodriguez K Benitez JR Rodriguez M Boelaert M Costs of dengue prevention and incremental cost of dengue outbreak control in Guantanamo, Cuba Trop Med Int Health 2012 17 1 123 132 10.1111/j.1365-3156.2011.02881.x 21906216

[B14] Beauté J Vong S Cost and disease burden of dengue in Cambodia BMC Public Health 2010 10 521 10.1186/1471-2458-10-521 20807395 PMC2941493

[B15] Guzmán MG Triana C Bravo J Kourí G The estimation of the economic damages caused as a consequence of the epidemic of hemorrhagic dengue in Cuba in 1981 Rev Cubana Med Trop 1992 44 1 13 17 1344680

[B16] Valdés LG Mizhrahi JV Guzmán MG Economic impact of dengue 2 epidemic in Santiago de Cuba, 1997 Rev Cubana Med Trop 2002 54 3 220 227 15846947

[B17] Añez G Balza R Valero N Larreal Y Economic impact of dengue and dengue hemorrhagic fever in the state of Zulia, Venezuela, 1997–2003 Rev Panam Salud Publica 2006 19 5 314 320 16805973 10.1590/s1020-49892006000500004

[B18] Armien B Suaya JA Quiroz E Sah BK Bayard V Marchena L Clinical characteristics and national economic cost of the 2005 dengue epidemic in Panama Am J Trop Med Hyg 2008 79 3 364 371 18784227

[B19] Canyon DV Historical analysis of the economic cost of dengue in Australia J Vector Borne Dis 2008 45 3 245 248 18807382

[B20] Okanurak K Sornmani S Indaratna K The cost of dengue hemorrhagic fever in Thailand Southeast Asian J Trop Med Public Health 1997 28 4 711 717 9656390

[B21] Suaya JA Shepard DS Siqueira JB Martelli CT Lum LC Tan LH Cost of dengue cases in eight countries in the Americas and Asia: a prospective study Am J Trop Med Hyg 2009 80 5 846 855 19407136

[B22] Von Allmen SD López-Correa RH Woodall JP Morens DM Chiriboga J Casta-Velez A Epidemic dengue fever in Puerto Rico, 1977: a cost analysis Am J Trop Med Hyg 1979 28 6 1040 1044 507281 10.4269/ajtmh.1979.28.1040

[B23] Kay BH Tuyet Hanh TT Le NH Quy TM Nam VS Hang PV Sustainability and cost of a community-based strategy against aedes aegypti in northern and central Vietnam Am J Trop Med Hyg 2010 82 5 822 830 10.4269/ajtmh.2010.09-0503 20439962 PMC2861387

[B24] Baly A Toledo ME Boelaert M Reyes A Vanlerberghe V Ceballos E Cost effectiveness of aedes aegypti control programmes: participatory versus vertical Trans R Soc Trop Med Hyg 2007 101 6 578 586 10.1016/j.trstmh.2007.01.002 17368696

[B25] Vazquez-Prokopec GM Chaves LF Ritchie SA Davis J Kitron U Unforeseen costs of cutting mosquito surveillance budgets PLoS Negl Trop Dis 2010 4 10 e858 10.1371/journal.pntd.0000858 21049010 PMC2964299

[B26] Taliberti H Zucchi P Direct costs of the dengue fever control and prevention program in 2005 in the city of são Paulo Rev Panam Salud Publica 2010 27 3 175 180 10.1590/S1020-49892010000300004 20414506

[B27] Carrasco LR Lee LK Lee VJ Ooi EE Shepard DS Thein TL Economic impact of dengue illness and the cost-effectiveness of future vaccination programs in Singapore PLoS Negl Trop Dis 2011 5 12 e1426 10.1371/journal.pntd.0001426 22206028 PMC3243704

[B28] Lee BY Connor DL Kitchen SB Bacon KM Shah M Brown ST Economic value of dengue vaccine in Thailand Am J Trop Med Hyg 2011 84 5 764 772 10.4269/ajtmh.2011.10-0624 21540387 PMC3083745

[B29] Shepard DS Suaya JA Halstead SB Nathan MB Gubler DJ Mahoney RT Cost-effectiveness of a pediatric dengue vaccine Vaccine 2004 22 9–10 1275 1280 15003657 10.1016/j.vaccine.2003.09.019

[B30] Alphey N Alphey L Bonsall MB A model framework to estimate impact and cost of genetics-based sterile insect methods for dengue vector control PLoS One 2011 6 10 e25384 10.1371/journal.pone.0025384 21998654 PMC3187769

[B31] Suaya JA Shepard DS Chang MS Caram M Hoyer S Socheat D Cost-effectiveness of annual targeted larviciding campaigns in Cambodia against the dengue vector aedes aegypti Trop Med Int Health 2007 12 9 1026 1036 10.1111/j.1365-3156.2007.01889.x 17875014

[B32] McConnell KJ Gubler DJ Guidelines on the cost-effectiveness of larval control programs to reduce dengue transmission in Puerto Rico Rev Panam Salud Publica 2003 14 1 9 16 10.1590/S1020-49892003000600003 12952602

[B33] Orellano PW Pedroni E Cost-benefit analysis of vector control in areas of potential dengue transmission Rev Panam Salud Publica 2008 24 2 113 119 19062602 10.1590/s1020-49892008000800005

[B34] Luz PM Vanni T Medlock J Paltiel AD Galvani AP Dengue vector control strategies in an urban setting: an economic modelling assessment Lancet 2011 377 9778 1673 1680 10.1016/S0140-6736(11)60246-8 21546076 PMC3409589

