# Supplementary figures and images for: Cost of dengue outbreaks: literature review and country case studies
Source: BMC Public Health. 2013 Nov 6;13:1048. doi: 10.1186/1471-2458-13-1048 (PMC4228321; doi:10.1186/1471-2458-13-1048)

## Slide 1
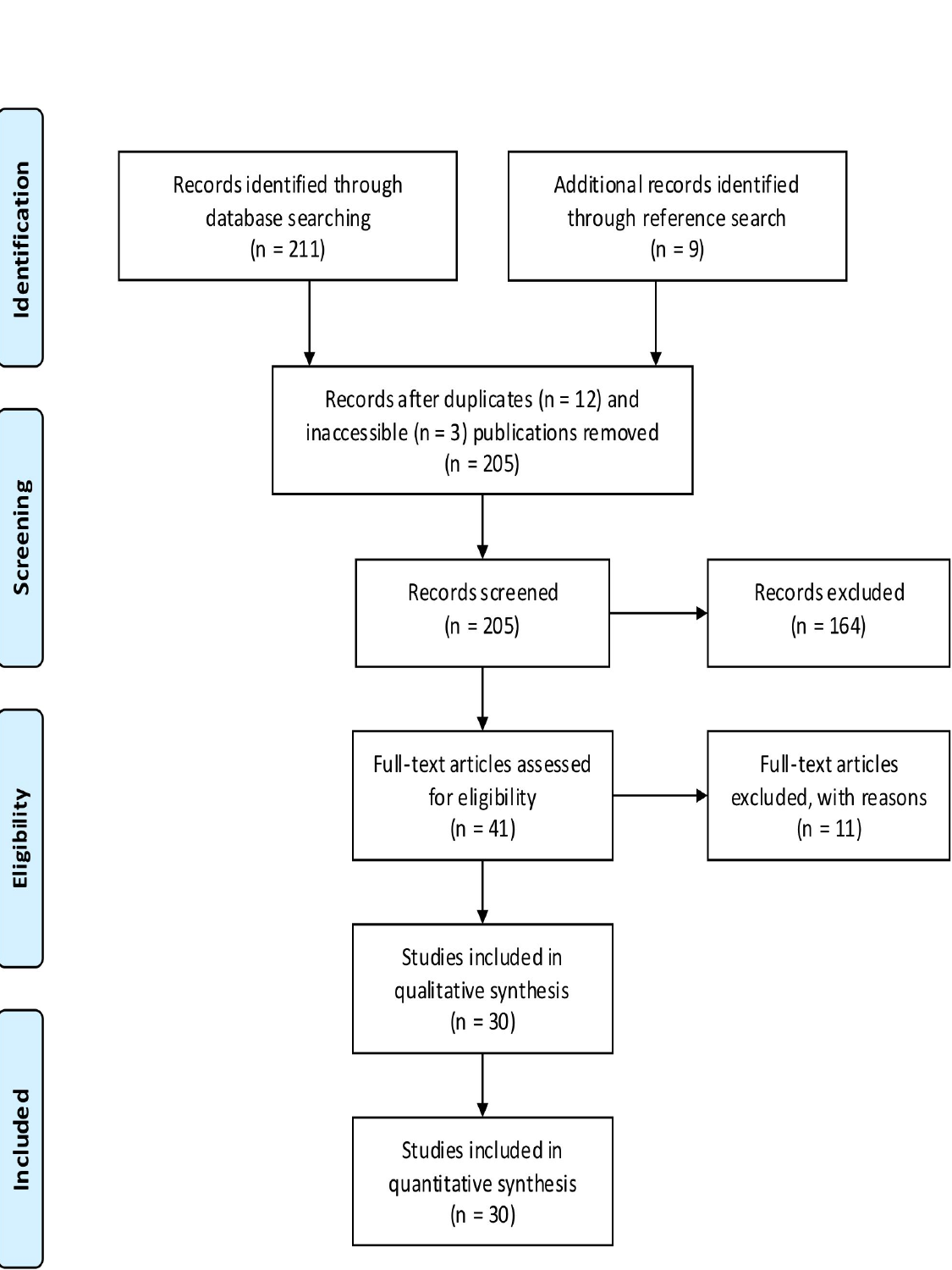

Supplement: Additional file 1: Figure S1 — PRISMA Flow-chart of review process. [file 1471-2458-13-1048-S1.pptx]
