# Supplementary material for: Cost of dengue outbreaks: literature review and country case studies
Source: BMC Public Health. 2013 Nov 6;13:1048. doi: 10.1186/1471-2458-13-1048 (PMC4228321; doi:10.1186/1471-2458-13-1048)
Supplement: Additional file 3: Table S2 — Extraction matrix of selected and reviewed literature. [file 1471-2458-13-1048-S3.docx]

**Table S2: Extraction matrix of selected and reviewed Literature**

| **First Author, Publication Year, Reference, Evidence** | **1) Countries, 2) Cities, 3) Study Period** | **1) Interventions, 2) Control, 3) Randomized** | **1) Perspective, 2) Outcome** | **1) Subjects, 2) Inclusion, 3) Exclusion Criteria, 4) Total cases** | **1) Cost Type, 2) Cost Year, 3) Currency, 4) Cost Horizon** | | **1) Costs** | **1) Effect Type, 2) Effect Year, 3) Effect Horizon** | **1) Effects** | **Remarks** |
| --- | --- | --- | --- | --- | --- | --- | --- | --- | --- | --- |
| Alphey (2011), (30), UE | 1) & 2) Simulation model  3) Cost data based on Suaya (2009) | 1) Release of Insects Carrying a Dominant Lethal to avert case (RIDL)  2) No Vector Control  3) Simulation model | 1) More economic arguments than societal  2) Cases averted by vector control | 1) Population size: 2.000.000 or 10.000  2) & 3) Simulation model  4) 48 to 49.000 annual cases in a population of 2 million without vector control | 1) SIT facility cost and operational costs for the intervention group  2) Model  3) US$  4) 2008 US$ using GDP chained Price index | | In Millions US$  **5 years program:**  2 million pop: Total (construction + operational) costs: range is 0.342–1.109 for release 1 and 2.878–10.549 for release 10  10 000pop: Total (construction + operational) costs: range is 0.002–0.006 for release 1 and 0.018–0.056 for release 10  **10 years program;**  2 million pop: Total (construction + operational) costs: range is 0.432–1.960 for release 1 and 3.775–19.059 for release 10  10 000pop: Total (construction + operational) costs: range is 0.002–0.009 release1 and 0.022–0.098 release 10 | 1) Cases averted  2) Model  3) Program runs for 5 or 10 years | Cases averted in 5 years for 2000000 pop: 239,816 for release 1 and 243 532 for release 10  Cost per cases averted:  5y:  2millionpop: US$2.83 (1) vs US$25.65 (10)  10000pop:US$3.00 (1) vs US$29.11 (10)  10y:  2millionpop: US$2.26 (1) vs US$21.33 (10)  10000pop: US$2.34 (1) vs US$23.10 (10) | "The ICER between release ratio 1(low) and 10 (high) is calculated in the paper!  The ICERs shown in Table 4 range from US$939 to US$2524, which exceed the overall average societal cost per infection US$86–190. So, using our measure (cases averted during the 5 or 10 year period) the extra cost of the higher release ratio is not worth the additional health effects obtained by disrupting virus transmission more quickly" |
| Anderson (2007), (6), I | 1) Thailand  2) Kamphaeng Phet province in northern Thailand  3) 1998-2002 | 1) –  2) No dengue fever  3) prospective cohort study in primary schools in thailand | 1) –  2) DALY and cost/patient | 1) Children at primary school  2) Attendance at one of the 12 chosen primary schools, enrolment in first to sixth grades thereafter, and parental written informed consent, age: 5-15  3) Planning to move outside the study area within the first 12 months and history of thalassaemia  4) 2119 children recruited | 1) Medication and clinic costs + associated expense of lost income  2) 1998-2002  3) converted to US$ with reported exchange rate for each year  4) 1998-2002 | | Mean cost of illness : $16.59  [non-hospitalized: $10.15 (95% CI 1.10–18.14) and hospitalized: $31.79 (95% CI 24.35–44.38)] | 1) DALYs  2) 1998-2002  3) 1998-2002 | 4653 (SD 3580) DALYs / million / year | They do a comparison to non-dengue fever cases. |
| Anez (2006), (17), IV | 1) Venezuela  2) Estado de Zulia  3) 01.01.1997-31.12.2003 | 1) –  2) –  3) No | 1) –  2) Direct and indirect costs | 1) DF, DHF,/SCD  2) Patient registry  3) no given  4) 30251 DF and 3606 DHF/SCD | 1) direct and indirect cost (lost days of work)  2) 1997 to 2003  3) US$  4) 1997 to 2003 | | Direct cost: 132.042,30 US$ (Outpatients), 342.209,40 US$ (Inpatients)  Indirect cost: 873.825,84 US$ | 1) –  2) –  3) - | 1) - | Dengue peak: 2002, DH/SCD peak 1999 – the cost for hospitalized patients (94,90 US$) were calculated for 2004 and applied to other years. They are not identical to cost figures of other studies. Outpatient costs (3,90 US$) do converge with other studies. |
| Armien (2008), (18), IV | 1) Panama  2) –  3) Oct 14, 2004 to Sep 8, 2005 | 1) –  2) Prospective non-comparative observational study  3) randomized patient interviews based on lab-confirmed batches (1 out of 3 to 5) | 1) Societal  2) impact of dengue on patients' health, use of medical services, schooling, work productivity, leisure time, out-of-pocket spending, and income loss. Clinical characteristics assessed duration of fever and illness, symptoms, signs of disease, perceived severity and quality of life | 1) Lab confirmed dengue patients  2) dengue confirmed serology or virology, ambulatory care-seekers at a public health facility, patients address known, informed consent  3) –  4) 162 patients all ages (N = 23 Children / 107 Adults) | 1) National cost associated with 2005 dengue epidemic: a) dengue cases b) national dengue control efforts (surveillance, laboratory, vector control)  2) 2005  3) US$  4) 2005 dengue outbreak | | 1) Non-fatal dengue: 11.269.187 US$ - Fatal-dengue: 558.680 US$ - National vector control, surveillance and lab cost/capita 1,56 US$ - National total dengue cost/capita 5,22 US$ | 1) Quality of Life using the World Health Survey (pain and discomfort, mobility, interpersonal activities, sleep and energy, affect, self-care, cognition, vision) and Euroqol VAS  2) 2005  3) during illness | 1) EQ-5D VAS score: Children 35,2 (20,8) - Adults 32,9 (21,3) | Outbreak costs – detailed cost tables |
| Baly (2007), (24), II | 1) Cuba  2) Santiago de Cuba  3) 2001-2002 | 1) Community-based intervention integrated in vertical programme  2) routine vertical vector control programme  3) 20 Family medical practices randomly selected for intervention +20 control | 1) a) Health System Provider (Provider Cost of Vector Control + Contribution from primary Healthcare services) b) Provider cost of vector control only c) Community perspective (unpaid time) d) society (health system+community)  2) incremental costs per number of foci; intervention more cost-effective from viewpoint of society, health system and vertical program perspective. | 1) larval breeding sites  2) –  3) –  4) 2.400 houses (intervention) 2.600 houses (control) | 1) Labour costs, consumables (for larval control and spraying), costs of training and social communication, operating expenses (food, travel, fuel etc.), capital costs  2) 2000, 2001, 2002  3) US$  4) 2000-2002 | | Total cost vertical control program (13 US$ / Inhabitant (2000); 15 US$ / Inhabitant (2001); 24 US$ / Inhabitant (2002)) | 1) entomological indicators (number of foci, house index, changes in location of main breeding sites) - behavioral change indicators (number of covered tanks, unprotected containers and containers protected by larvicide)  2) 2000, 2001, 2002  3) 2000-2002 | Reduction in no. of foci: intervention 459; control 467 | Study was designed in light of dengue outbreak in 2001 in Havana |
| Baly (2011), (12), UE | 1) Venezuela and Thailand  2) Trujilo and Laem Chabang  3) 2007 | 1) Distribution of Insecticide-treated curtains / Cost of Routine Ae. aegypti Control Program  2) –  3) - | 1) Societal perspective  2) 2,14 US$ / Household in Venezuela - 1,89 US$ / household in Thailand | 1) Households  2) Trujillo 10 clusters, 400 houses from 18 districts with at least 4/1000 habitants dengue notification rates. / In Thailand 22 Clusters of 100 houses randomly selected in 4 town districts  3) –  4) 4.000 houses in Venezuela and 2.200 houses in Thailand | 1) Recurrent costs (personnel, supplies, materials, operation and maintenance, utilities, rent of transport or infrastructure) - capital costs (ITC, depreciation of vehicles, equipment).  2) 2007  3) US$  4) Yearly costs | | 2,14 US$ / Household in Venezuela - 1,89 US$ / household in Thailand | 1) –  2) –  3) - | 1) - |  |
| Baly (2012), (13), UE | 1) Cuba  2) Guantanamo  3) 2006 | 1) –  2) Non-endemic period  3) No | 1) Societal  2) Entomological indicators: monthly number of A. Aegypti-positive houses, Health care services output: number of febrile cases detected, patients hospitalized, lab test performed, hospital indicators (number of admissions because of dengue, average length of stay, number of discharges, number of diagnostic tests performed) | 1) Data gained by reviewing routine reports and registers for entomological, health care and programme output. Data for hospital indicators collected: accountancy department of the general  provincial hospital. Data for intersectoral activities during the outbreak: Provincial Centre for Hygiene and Epidemiology. Semi-structured interviews with: 100 family doctors, nurses and vector control personnel, 200 household members, 100 community leaders. Out-of-pocket and productivity losses: 1/3 adult cases discharged between Sept. and October 2006  2) –  3) –  4) 3549 suspected, 2937 confirmed cases of dengue | 1) Direct and indirect: - recurrent costs (salaries, supplies & materials (larvicides and insecticides, diagnostic tests, drugs, protective clothing, gloves, office materials) -operational costs (fuels, lubricants, vehicle, rent, per diems and food, spare parts, maintenance of equipment, vehicles and buildings) -utilities (electricity, water, telephone) capital means (portable fogging equipment, trucks for spatial spraying, other vehicles, laboratory equipment, furniture) - out-of-pocket and productivity losses  2) 2006  3) US$  4) 2006 | | VECTOR CONTROL= Jan-Jul: 1.67$ per month (p.m), per inhabitant (p.i) Aug-Dec: 1.88 p.m,p.i HEALTHCARE COSTS= 0.25$pm/pi (Jan-Jul) vs 1.6$ pm/pi (Aug-Dec) COMMUNITY level: average economic cost pm/pi 0.84 (jan-july) vs 2.48$ (aug-dec) TOTALCOST per hospitalized case: 296.60$ Insgesamt: 6.05$pm/pi (aug-dec) vs 2.76 (jan-july) | 1) -  2) –  3) - | 1) - | - |
| Beauté (2010), (14), UE | 1) Cambodia  2) Kampong Cham Province  3) 2006, 2007, 2008 | 1) Cost of Illness study  2) –  3) - | 1) Societal  2) Costs and DALYs | 1) Inhabitants of Kampong Cham Province (14.354 participants)  2) Living in one of 42 villages and suffering from DF, DHF, DSS  3) –  4) 2006: 76.933  2007: 404.165  2008: 12.1007 | 1) Medical Resources (direct treatment and direct control costs) and indirect costs  2) 2006-2008 (3% discount rate/year)  3) US$  4) 2006-2008 | | Per dengue case:  2006: 75$  2007:36$  2008: 27$  Total Costs:  2006: 5,771,079  2007:14,429,513  2008: 3,327,284 | 1) –  2) –  3) – | 1) - | DALY rate by year/ by 100,000 inhabitants:  2006: 40  2007: 116,6  2008: 24,3 |
| Canyon (2008), (19), V | 1) Australia  2) Whole Australian country  3) 1885-2008 | 1) Historical Analysis  2) –  3) – | 1) –  2) – | 1) Australian Population  2) –  3) –  4) 1885-1980: 1.819.340  >1980-2008: 35.000 | 1) indirect costs + vector control costs+ direct costs  2) –  3) AUS$  4) – | | Indirect costs:  prior to 1990: ~2billion  from 1990-2008:  41.3 million  Total costs: since 1990:  around 2.7 million $ per annum | 1) –  2) –  3) – | 1) - | - |
| Carrasco (2011), (27), UE | 1) Singapore  2) National  3) 2000-2009 | 1) Cost of Illness  2) –  3) – | 1) –  2) DALYs realted to D and DHF | 1) Singapore population  2) Reported cases to Ministry of Health  3) –  4) – | 1) Direct costs & indirect costs: reduction of work productivity, loss of schooling, increased need for caregivers  2) 2010  3) US$  4) 2010 | | Total economic costs from 2000 to 2009 were $0.91 billion using the human capital method or $0.85 billion using the friction cost method | 1) DALYs  2) –  3) – | DALYs per 100,000 population were 8.7 (5th and 95th percentiles of 8 and 10) when using constant symptomatic rates and 14 (5th and 95th percentiles of 13 and 16) when using age-dependent symptomatic rates | Cost-Effectiveness of Vaccination is evaluated as well |
| Clark (2005), (7), I | 1) Thailand  2) Kamphaeng Phet  3) 2001 | 1) –  2) –  3) Cluster sampling design | 1) –  2) Cost and DALYs | 1) Persons in Kamphaeng Phet hospitalized with laboratory-confirmed dengue virus infection in 2001  2) 2001  laboratory-confirmed dengue infection  3) –  4) 124.409 cases of dengue in Thailand in 2001, of which 204 cases were sampled to complete the surveys. | 1) Direct economic costs of hospitalization (transportation, lodging, food) and hospitalization costs incurred by the family + opportunity costs due to loss of productivity  2) 2001  3) Baht (but approx. US$ given)  4) 2001 | | Total costs: 24 US$ / Family | 1) –  2) –  3) – | - | Hospitalization for children < 12 y is free in Thailand!  DALYs lost per million population due to dengue: 427 |
| Damme (2004), (8), IV | 1) Cambodia  2) Banteay Meanchey  3) August 2001 (first survey)- May 2002 (second survey) - June 2002 (follow-up survey) | 1) Gain qualitative and semi- quantitative insight into out-of-pocket health expenditure through household surveys  2) –  3) – | 1) –  2) Out-of-Pocket Expenditure | 1) Households living in the area covered by the public hospital  2) –  3) –  4) First survey: 42 households  Second: additional 30 households  Follow-up: 26 households of the first survey | 1) Out-of-pocket expenditures  2) 2001-2002  3) Cambodian riel, Thai baht and US dollars (all converted to US$ using the exchange rates at the time of the survey)  4) 2001-2002 | | OOP- Payments:  Private providers only: US$103  Combination of private provider and public hospital: 32  Public hospital only: 8 | 1) Are the OOP-Payment leading to debts?  2) 2001-2002  3) 2001-2002 | After the treatment, 45 of 72 households (63%) were in debt.  After 1 year (follow-up survey): only 38% of them had been able to completely settle their debts | - |
| Garg (2008), (11), I | 1) India  2) -  3) 1996-2007 Review; Sep-Nov 2006 single hospital data; 2006 for register data | 1) –  2) –  3) – | 1) Review of Publications; data on hospitalized patients at a single hospital during the 2006 dengue epidemic; data on dengue cases and deaths during the 2006 dengue epidemic in India  2) To estimate economic burden of dengue within the private and state health sectors in India | 1) -  2) -  3) -  4) - | 1) -  2) -  3) -  4) - | | 1) - | 1) –  2) –  3) - | 1) – | Dengue epidemic 2006 |
| Guzman (1992), (15), IV | 1) Cuba  2) Cuba  3) 1981 | 1) Estimation of cost of the Cuban epidemic in 1981  2) -  3) - | 1) –  2) Costs | 1) Cases of DF/DHF during epidemic in 1981  2) –  3) –  4) 344.203 (10.312 severe, 158 deaths) | 1) Hospitalization expenses, assistance in emergency units, social security, expenses in out-patient treatment, goods not produced and expenses in the anti-vector campaign  2) 1981  3) US$  4) 1981 | | hospitalization expenses: 38.796.316  assistance in emergency units: 1,290,854  social security:4,724,040  expenses in out-patient treatment  goods not produced : 14,318,760  expenses in the anti-vector campaign:43,000,000  Meds for ambulatory patients: 1,021,673  TOTAL: 103,151,643 | 1) –  2) –  3) – | 1) - | The epidemic could have been controlled in about 4 months. |
| Harving (2007), (9), IV | 1) Vietnam  2) Ho Chi Minh City  3) Aug 2005 – Nov 2005 | 1) –  2) –  3) – | 1) Patients/  Caretakers  2) Measuring the economic consequences of having a relative admitted with DHF | 1) Children with DHF  2) Age: 0-15 years; clinically diagnosed as having DHF according to guidelines; diagnosis confirmed by ELISA test  3) Insufficient data obtained in the interview; other chronic diseases  4) 175 | 1) Direct and indirect costs  2) 2005  3) US$  4) 1 DHF episode | | 61,31 US$ per case | 1) -  2) –  3) - | 1) - |  |
| Huy (2009), (10), IV | 1) Cambodia  2) 16 villages in 3 districts of Kampong Cham Province  3) May-Dec 2006 | 1) 2 parts: estimating the incidence (surveillance study) and case control to compare dengue specific costs and health-seeking behaviour  2) Non-dengue fever control assigned to each acute dengue case  3) Matched pairs | 1) –  2) Costs, health-seeking behaviour | 1) Study cohort  2) <15ys  3) >15ys  4) Study cohort=6.694  Lab confirmed=89  Case-Control=last 30 | 1) Direct and indirect costs  2) 2006  3) US$  4) 2006 | | 1) Average total cost of illness to the household:  - for dengue fever: 31,50$  -non-dengue febrile illness: 27,20$ | 1) Duration of illness, health status reported, hospitalization  2) 2006  3) 2006 | 1) No significant difference between dengue-fever and non-dengue fever patients regarding reported health status (reported as bad or very bad in 14% vs 19%), duration of fever (7.5 days vs 7.1 days) or days of hospitalization (4.8 days and 4.8 days) | The cost study survey questionnaire is available under:  <http://www.biomedcentral.com/content/supplementary/1471-2458-9-155-S1.pdf> |
| Kay (2010) (23), UE | 1) Vietnam  2) Xuan Truong and Ninh Xuan  3) 2007, 2008 | 1) Cross-sectional study to determine level of residual community dengue-control activity, KAP (knowledge, attitude, practice)  2) North: four communes where some dengue education and control activities had been implemented under the National Dengue Control Program (NDCP) from 2000 in Trung Dong, Minh Tan, Dai Thang, and Lien Minh (North Control Communes [NCC]  Central: Neighboring Ninh Binh commune (no formal dengue control=Central Control Commune [CCC]  3) Systematic random samples of 100 houses were chosen for entomological and KAP survey subjects in each commune (in total, N = 300 and 200, respectively)) | 1) –  2) Comparison between effects during intervention and several years after | 1) Households in Nam Dinh Province (North) and Ninh Xuan in Khanh Hoa Province (central Vietnam)  2) Households in which entomological surveys were conducted  3) People aged < 18 years old and people without the cognitive ability to participate in the interview  4) North:  Project commune (Xuan Phong) 10.100  Extended commune (Tho Nghiep) 12.000  NDCP communes (N = 4) 40,463  Central:  Project commune (Ninh Xuan) 11.110  Untreated commune (Ninh Binh) 10.856 | 1) Direct costs: stipends for collaborators and commune-management committees; supplies for schools and collaborators.  Indirect costs: opportunity costs were estimated based on reported incomes that would have otherwise been earned by the collaborators  2) –  3) Vietnames Dong (VND) converted in International Dollars (I$)  4) – | | NPC= 6,134 I$ with an additional 10% used for start-up costs incurred in the first year  NEC=10,736 I$ with an additional 1% used for start-up costs incurred in the first year  CPC= 3,098 I$ | 1) 13 criteria grouped under: maintenance of health benefits achieved through the initial project, continued delivery of project activities, long-term capacity building in the recipient community. Rated on a 1-5 scale  2) 2007/2008  3) 2000/03-2007 (north) 2003-2008 (central) | 1) KAP was > in NPC & NEC compared with NCC  Project activities were best delivered by NPC and NEC compared with control communes.  In NPC Mesocyclops prevalence 2007 was not different to 1999, but > than NEC  Long-term capacity building was strongly maintained in NPC and NEC compared with NCC.  Health benefits were superior in CPC compared with CCC  Householders’ knowledge of dengue vector behavior, including larval habitats and resting places, was maintained.  Long-term capacity building at CPC was not as strong as at NPC and NEC. | Reason for difference between north and central: No expansion from the original project commune occurred, which is in contrast to the north. |
| Lee (2011), (28), UE | 1) Thailand  2) –  3) – | 1) Vaccination  2) No Vaccine  3) Decision tree | 1) Societal  2) Cost-Effectiveness of Vaccine | 1) Parameters of population of Thailand  2)  3)  4) 1,000 individuals through the model 1,000 times equating to one million realizations. | | 1) Clinic visit, medical visit, vaccine minor side effect  2) 2010  3) US$  4) – | Clinic visit $11.09  Hospital visit :  DF $34.74  DHF $42.71  Vaccine minor side effect $0.31 | 1) –  2) 2010-2011  3) 2010 | 1) Vaccinating was highly cost-effective (ICER < 4,289) for all scenarios up to a $60 vaccination price point  Vaccination remained cost-effective (4,289 > ICER < 12,868) through vaccination costs of $200, and remained cost-effective at vaccination price points of $400 if the vaccine efficacy was at least 75% and infection incidence was ≥ 9% | Markov Model used for computation |
| Luz (2011), (34), UE | 1) Brazil  2) Rio de Janeiro  3) - | 1) 42 different insecticide-based vector control strategies (adult/larval stage targeted; varying efficacies (high/med/low); yearly application frequency (1-6)  2) No vector control  3) Dynamic model of dengue transmission is used, including seasonality and population genetics of insecticide-resistance. (Mathematical model predicting DALYs) | 1) Societal  2) DALYs | 1) Demographic dynamics of Population of Rio de Janeiro are incorporated into the model  2) -  3) -  4) - | | 1) Direct and indirect costs  2) 2009  3) US$  4) - | 1) – | 1) DALYs  2) 5 years (model)  3) 5 years (model) | 1) For 5 ys: No intervention--> 1133 DALYs lost per million population |  |
| McConnell (2003), (32), IV | 1) Puerto Rico  2) –  3) 1983-1989 (data used) | 1) Larval control  2) No larval control  3) - | 1) –  2) - | 1) –  2) –  3) –  4) – | | 1) Direct & indirect cost and intervention costs  2) 1983-1989  3) US$  4) - | 1) Cost of one clinical visit 96 US$  Cost of one hospital visit 1.389 US$ | 1) -  2) -  3) - | 1) - | - |
| Okanurak (1997), (20), IV | 1) Thailand  2) Bangkok + Suphan Buri + Don Chedi District  3) Jul-Aug 1994 | 1) Collection of cost data of dengue haemorrhagic fever: interviews using closed and open-ended questionnaires  2) -  3) - | 1) Societal  2) Cost of illness | 1) DHF patients  2) DHF Patient in one of the 3 chosen Hospitals: Children's hospital (Bangkok) and Suphan Buri Provincial Hospital and Don Chedi Community Hospital (Suphan Buri Province)  3) –  4) 184 patients  -72 children's hospital  -99 from Suphan Buri Provincial Hospital  -13 from Don Chedi Community hospital | | 1) Direct patient costs and opportunity costs (absence from work: patients but also main (parents) and assistant (relatives helping) caretakers  2) 1994  3) US$  4) 1994 (Jul-Aug) extrapolation to the total year of 1994 | 1) Total Costs  Adults:  161.49 US$ in Bangkok  138.02 US$ in Suphan Buri  Children:  118.29 US$ (Bangkok)  102.82 US$ (Suphan Buri) | 1) -  2) -  3) - | 1) - | Ranges included in the study. |
| Orellano (2008), (33), II | 1) Argentina  2) Clorinda  3) Jan-Apr 2007 | 1) Vector control  2) –  3) Cost-Benefit Analysis | 1) Societal  2) Cost Benefit Analysis | 1) Clorinda population  2) –  3) –  4) - | | 1) Direct and indirect cost  2) 2007  3) US$  4) Jan-Apr 2007 | Without intervention:  TC=106.724 US$, AC=2,27 US$  With Intervention:  TC=378372 US$  AC=8,05 US$ | 1) Averted work days lost  2) 2007  3) Jan-Apr 2007 | Without Intervention: 0 With Intervention: 575.251 | Sensitivity analysis shows that the intervention is dominant with an incidence of more than 29:1000 DF |
| Shepard (2011), (3), UE | 1) The Americas  2) Nationwide  3) Review data from 2000 to 2007 | 1) Review  2) –  3) – | 1) Societal  2) Review for an estimation of economic and disease burden based on: reported dengue case / degree of underreporting / direct and indirect cost / DALY burden / demographic information | 1) American population affected be dengue fever/DHF  2) Systemic literature review, PAHO, US Census Bureau  3) –  4) Annual Average of dengue cases in the Americas for 2000-2007: 5.6 Million (adjusted for underreporting) | | 1) Direct and indirect cost  2) Updated to 2010 US$  3) US$ and IS  4) Adjusted from the different years (2005 and 2009 e.g.) to 2010  Productivity losses: discounted life years lost / GDP per capita using 3 % discount rate | 1) Annual total cost of dengue in the Americas: 2.1 billion US$ | 1) DALYs  2) Data from 2000-2007 discounted and adjusted to 2010  3) 2010 | DALYs lost/year for the Americas: 72.277  DALYs per million inhabitants: 50-131 | There are large differences among the countries regarding costs and DALYs. |
| Shepard (2004), (29), I | 1) South East Asia  2) –  3) – | 1) Vaccination of cohort group  2) No Vaccination  3) - | 1) Societal  2) Cost-effectiveness of vaccination measured in DALYs | 1) Cohort of 11.6 million children in SE Asia  2) -  3) -  4) - | | 1) Treatment costs estimated from Thailand data (ambulatory visits, hospitalization, medication, travel expenses, parent's time) but adjusted for wage rates and GNI per country, vaccination costs, laboratory costs  2) 1999  3) US$  4) Lifetime of cohort group, 3% discount rate | 1) Baseline cost of treatment (per 1.000 inhabitants/year): 99 US$ | 1) DALYs  2) 1999  3) Lifetime of cohort group (expectancy in SE Asia 66 years) | Baseline for SE Asia: 0.42 DALYs/1000 inhabitants  DALYs saved by vaccination: 0.34 /1000 population per year  Cost/DALY saved: 50$ | Total costs and total DALYs are calculated as well! |
| Suaya (2009), (21), I | 1) Brazil, El Salvador, Guatemala, Panama, Venezuela, Cambodia, Malaysia, Thailand  2) Goiana City(Brazil), San Salvador (El Salvador), Guatemala City (Guatemala), Panama Province (Panama), Coro City (Venezuela), Takeo Province (Cambodia), The Klang Valley (Kuala Lumpur Area, Malaysia), Khon Kaen Province (Thailand)  3) Recruitment period: September 2004 through January 2007 | 1) Cost-of-illness Study so no intervention/control group  2) Cost-of-Illness Study so no Intervention/Control Group  3) No | 1) Cost of dengue cases  2) Unit of analysis: dengue case | 1) Dengue patients in 8 countries in major provincial or national reference public hospitals, in 6 sites also ambulatory, in Brazil also private facilities  2) Patients with febrile illness meeting the WHO clinical case definition for dengue; In Panama and Venezuela: only laboratory-confirmed cases included. 8 countries: with children < 15y; 5 countries with also adults  3) –  4) 1695 dengue cases in 8 countries | | 1) Direct (medical and non-medical) and indirect costs  2) 2005  3) International Dollars (I$), adjusted for Purchasing Power Parity  4) Economic costs: use of official 2001-2005 reports | 1) Great variation within different countries: mean unweight cost per case: I$ 514 for ambulatory patients and I$1394 for hospitalized patients | 1) –  2) -  3) - | 1) - | Payment of Health Care providers completely different in the different countries. Furthermore: mean cost of dengue reported case weighted by official numbers of deaths and cases by setting was I$759 in America, vs I$2005 in Asia! |
| Suaya (2007), (31), I | 1) Cambodia  2) Phonm Penh and Province of Kandal  3) 2001-2005 | 1) Annual larviciding campaign targeting medium to large-size water storage containers located at households in densely-populated areas. 2 rounds done in 2001,2002 and 2005 [April+July] only one [at time of outbreak] done in 2003 and 2004  2) Control Areas: Areas elsewhere in Cambodia than Phnom Penh or Province of Kandal  3) No | 1) Public sector and societal perspective  2) dengue hospitalized and ambulatory cases averted, dengue deaths averted, DALYs saved from reduction in morbidity and mortality | 1) Population of Cambodia divided in 2 different regions: intervention area: KK&P and control areas: everything else  2) –  3) –  4) Intervention group: 2.9 million people (23% of population in Cambodia) | | 1) Gross cost of intervention, savings in medical care, net of cost of annual larviciding campaigns  2) 2005  3) US$  4) 2005 | 1) Gross Cost in public sector larviciding campaign: 567800$/year [0.2$ per person living in KK&P]  Total savings in public sector= 255.586$  Net-Cost/year: 312.214$ [0.11$ p.P in KK&P] | 1) DALYs  2) 2005  3) 2005 | 1) Calculated weighted annual average: 997.1 total DALYs saved in the intervention area | 1) CE Ratio: 313$/DALY saved from public sector perspective  CE Ratio: 37$/DALY saved from societal perspective |
| Taliberti (2010), (26), UE | 1) Brasilien  2) Sao Paulo  3) 2005 | 1) Vector control  2) –  3) – | 1) –  2) – | 1) Population of Sao Paulo (10.927.985 Inhabitants)  2) Inhabitant of Sao Paulo  3) –  4) not given | | 1) Direct cost  2) 2005  3) US$  4) 2005 | 12.486.941,34 $ (direct cost)  (10927985 Inhabitants in Sao Paulo)= US$ 1,14 per capita | 1) -  2) -  3) - | 1) - | - |
| Valdes (2002), (16), IV | 1) Cuba  2) Santiago de Cuba  3) 1997 | 1) Isolation of patients  2) –  3) – | 1) –  2) - | 1) Dengue patients  2) Lab-confirmed  3) -  4) 17.926 cases | | 1) Direct and indirect cost  2) 1997  3) US$  4) 1997 | Hospitalization: 1.918.617 US$  Vector control:  7.787.500 US$  Lab: 38.222,60 US$  Total Cost:  10.251.539,80 US$ | 1) -  2) -  3) - | 1) - | - |
| Vazquez-Prokopec (2010), (25), UE | 1) Australia  2) City of Cairns, Queensland  3) Case study with a combination of mathematical model + cost analysis with data from 2003 and 2009 dengue fever outbreaks | 1) Surveillance system  2) Absence of control surveillance system  3) - | 1) –  2) Impact of delayed control responses to dengue epidemics | 1) Epidemiologic data of Cairns in Australia  2) –  3) –  4) - | | 1) Direct and indirect cost  2) 2003-2009  3) US$  4) 2003-2009 | 1) Cost of active surveillance: 0.15 (2003) and 1.1 (2009) million US$  Response to the same outbreaks 4–6 weeks later: cumulative costs of and 2009 outbreaks that are 86 [(or US$ 13 million (2003)]; and 346 [(or US$382 million (2009)] times as high | 1) Cost reduction  2) 2003-2009  3) 2009 | 1) delayed reaction to both Cairns dengue outbreaks would have resulted in drastically escalated total costs of up to US$ 382 million |  |
| Von Allmen (1979), (22), IV | 1) Puerto Rico  2) Whole country  3) Jul-Dec 1977 | 1) Calculation of Costs  2) –  3) - | 1) –  2) Costs | 1) Population of Puerto Rico  2) –  3) –  4) 196.652 (lower limit) / 584.354 (upper limit) | | 1) Direct and indirect cost  2) 1977  3) US$  4) 1977 | Direct costs: 2,375,876-4,665,149  Indirect: 3,651,329 - 10,894,247  Total costs: 6 - 15.6 Million US$ | 1) –  2) –  3) – | 1) - | hospital costs of complicated cases not included in calculation |
